# Mupirocin Promotes Wound Healing by Stimulating Growth Factor Production and Proliferation of Human Keratinocytes

**DOI:** 10.3389/fphar.2022.862112

**Published:** 2022-04-11

**Authors:** Danielle Twilley, Oleg Reva, Debra Meyer, Namrita Lall

**Affiliations:** ^1^ Department of Plant and Soil Sciences, Faculty of Natural and Agricultural Sciences, University of Pretoria, Pretoria, South Africa; ^2^ Department of Biochemistry, Genetics and Microbiology, Faculty of Natural and Agricultural Sciences, Centre for Bioinformatics and Computational Biology, University of Pretoria, Pretoria, South Africa; ^3^ Department of Biochemistry, Faculty of Science, University of Johannesburg, Johannesburg, South Africa; ^4^ School of Natural Resources, College of Agriculture, Food and Natural Resources, University of Missouri, Columbia, MO, United States; ^5^ College of Pharmacy, JSS Academy of Higher Education and Research, Mysuru, India; ^6^ Bio-Tech Research and Development Institute, University of the West Indies, Kingston, Jamaica

**Keywords:** mupirocin, human keratinocytes, wound healing, cell proliferation, human growth factors

## Abstract

Mupirocin has been reported for its role in the treatment of infected wounds through its antibacterial activity, however the role of mupirocin in promoting wound healing via alternative mechanisms has not been extensively evaluated. This study aimed to evaluate the potential effect of mupirocin to promote wound healing, not only through its antibacterial activity but by increasing human keratinocyte proliferation and growth factor production. In the scratch assay, using human keratinocytes (HaCat), mupirocin (at 0.1 and 0.2 mM) significantly increased wound closure compared to the vehicle control. Cell viability, measured from the scratch assay, verified the increase in wound closure, where mupirocin at both concentrations showed higher cell viability compared to the vehicle control. In addition, mupirocin at 0.1 mM significantly stimulated the production of hepatocyte growth factor and M-CSF in HaCat cells, whereas at 0.2 mM, PDGF-AA and EPO were increased. The findings of this study suggest that mupirocin, which is commonly used as an antibacterial agent for the treatment of wounds, also facilitates the wound healing process by stimulating the proliferation of human keratinocytes and enhancing the production of several growth factors involved in wound healing. This is the first report on the effect of mupirocin on growth factors expressed by human keratinocytes as well as the stimulation of keratinocyte proliferation.

## 1 Introduction

**FIGURE 1:**
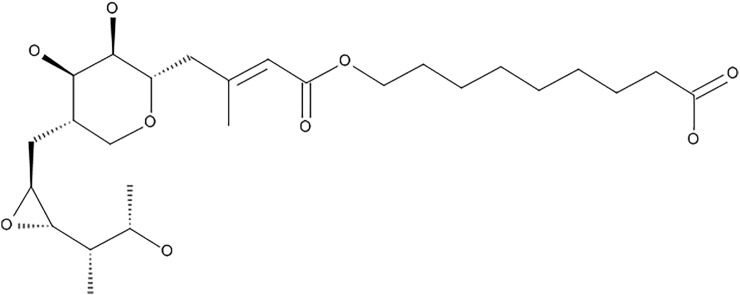
Chemical structure of mupirocin (pseudomonic acid A).

Mupirocin (pseudomonic acid A) (Figure 1), is a secondary metabolite produced by the Gram-negative soil bacterium, *Pseudomonas fluorescens* (Connolly et al., 2019). It is used as a topical antibiotic, which acts by binding to bacterial isoleucyl-tRNA synthetase (IleRS), thereby inhibiting protein synthesis (Abdulgader et al., 2020). It is used for the treatment of infections caused by pathogens such as streptococci and staphylococci strains, including methicillin-resistant strains (David et al., 2018). It is often used for the treatment of methicillin-resistant *S. aureus* (MRSA), which largely causes nosocomial bloodstream infections and is a major pathogen involved in wound infections (Conly and Johnston, 2002; Upreti et al., 2018; Abdulgader et al., 2020), however there is increasing evidence of mupirocin resistant staphylococci (Conly and Johnston, 2002). The growing resistance to mupirocin is largely due to its increased and uncontrolled use to treat infections. This has led to the development of low and high level resistance in *S. aureus*. Low level resistance is categorized to have MIC values ranging between 8 and 256 μg/ml, whereas high-level resistance is categorized as MIC values >512 μg/ml, however isolates with MIC values ranging between 128–256 μg/ml have been regarded as uncommon (Eltringham, 1997; Patel et al., 2009; Abdulgader et al., 2020). Mupirocin, is furthermore inactive against Gram-negative pathogens, due to its inability to target the membrane barriers, which has led to additional antibacterial mechanistic studies. Gilbert et al. (2005) developed small molecules namely, cationic steroid antibiotics (CSAs), which are able to bind to lipid A disaccharides located on the outer membranes of Gram-negative pathogens, thereby increasing membrane permeability which in turn sensitizes the bacterium to hydrophobic antibiotics, such as mupirocin. Gilbert et al. (2005), found that mupirocin had a minimum inhibitory concentration (MIC) of >85 μg/ml against *Escherichia coli*, which decreased to 1 μg/ml upon treatment of the bacteria with 1 μg/ml CSA (Gilbert et al., 2005).

Mupirocin has been previously reported to have wound healing effects, however this was primarily due to its antibacterial activity against wound associated bacteria. In a study by Golmohammadi et al. (2020), a nanohybrid system (mupirocin-SeNPs-CCH) was prepared using selenium nanoparticles (SeNPs) and mupirocin, which was entrapped using a chitosan-cetyltrimethylammonium bromide-based hydrogel (CCH). Using a rat diabetic wound model infected with mupirocin-methicillin-resistant *S. aureus* (MMRSA), it was reported that mupirocin-SeNPs-CCH significantly increased wound healing. In addition, the nanohybrid system decreased the MIC of mupirocin by 3-fold and played a significant role in wound contraction, angiogenesis, fibroblastosis, collagenesis, epidermis growth and proliferation of hair follicles. In a study by Pérez et al. (2021), the effect of photodynamic therapy (aPDT) and mupirocin was evaluated on a superficial skin infection (*S. aureus*) model in SKH-1 mice. Photodynamic therapy showed the most significant wound healing activity when compared to the use of mupirocin alone or mupirocin in combination with the photodynamic therapy, however the addition of mupirocin to the photodynamic therapy increased the antimicrobial activity. Verma and Kaushik, (2020) showed that copper nanoparticles, prepared from mupirocin, enhanced antibacterial activity against *S. aureus* through the sustained release of mupirocin over a 48 h period. Furthermore, Alizadeh et al. (2019), reported that copper nanoparticles (CuNPs) at a concentration of 1 µM and a size of 80 nm, were not toxic to human keratinocytes, fibroblasts and endothelial cells, enhanced the proliferation and migration of endothelial cells (HUVECs) and enhanced the expression of collagen 1A1. *In vivo* studies showed that these CuNPs were further able to increase wound healing in Wistar rats through the formation of granulation tissue and increased blood vessel formation.

Wound healing is a complex process which requires several cellular events and molecular interactions, of which growth factors play a major role (Barrientos et al., 2008). There are three major phases involved in wound healing: hemostasis and inflammation (inflammatory phase), granulation tissue formation (proliferative phase), and matrix formation and remodeling (remodeling phase) (Gushiken et al., 2021).The wound healing process is initiated by the secretion of cytokines, growth factors and other molecules which play crucial roles in the healing process. The inflammatory phase is associated with an influx of immune cells, such as neutrophils, monocytes and lymphocytes, which provide defense against microorganisms (through reactive oxygen species), phagocytosis of cell debris, and the production of cytokines and growth factors, which play a role in the proliferation phase. Keratinocytes initiate the proliferation stage of wound healing by migrating and proliferating at the wound edges, which is followed by the proliferation of dermal fibroblasts near the wound site, which in turn provide extracellular matrix components and ultimately result in wound contraction. Angiogenesis further aids wound healing, by forming new blood vessels, resulting in tissue granulation and eventually scar formation, which is accompanied by collagen synthesis. At each process within the wound healing phases, cytokines and growth factors play integral roles **(** Werner and Grose, 2003).

Although wound healing studies have focused mainly on the antibacterial potential of mupirocin, a few studies have reported the effect of mupirocin on inflammation and cell migration. Kamlungmak et al. (2021), showed that mupirocin stimulated the production of tumor necrosis factor (TNF)-α in RAW 264.7 cells. TNF-α is a critical cytokine involved in the inflammatory stage of wound healing. Ritsu et al. (2017) showed that TNF-α was detected immediately after a wound was created and increased in production until one day thereafter. Furthermore, the wound healing process in mice was delayed when treated with anti-TNF-α monoclonal antibodies and resulted in a decrease in the density of inflammatory cells and fibroblasts. On the contrary, administration of TNF-α significantly enhanced wound closure. This suggests the wound healing potential of mupirocin through an alternative mechanism other than through its antibacterial activity.

Therefore, in this study, we investigated the potential of mupirocin to enhance wound healing, not only through its previous reports on antibacterial against wound related pathogens, but by stimulating the proliferation of human keratinocytes and its modulatory effect on several growth factors associated with the wound healing process, which has not been previously reported.

## 2 Materials and Methods

### 2.1 Cell Culture

Human keratinocytes (HaCat), were donated by the Department of Human Biology, University of Cape Town, South Africa. The HaCat cells were maintained in DMEM, supplemented with 10% fetal bovine serum (FBS), 1% amphotericin B (250 μg/ml) and 1% antibiotics (penicillin at 100 U/mL and streptomycin at 100 μg/ml) (ThermoFisher Scientific, Johannesburg, South Africa), at 5% CO_2_ and 37°C. Cells were sub-cultured once an 80% confluent monolayer was observed using 0.25% trypsin-0.1% EDTA. Once the cells detached, the reaction was inhibited by the addition of supplemented DMEM. Cells were centrifuged at 980 rpm for 5 min, where after the cell viability was measured using the Countess automated cell counter (ThermoFisher Scientific, Johannesburg, South Africa) with 0.4% trypan blue solution.

### 2.2 Cytotoxicity

The cytotoxicity of mupirocin (purity ≥92%) (Sigma Chemicals Co., St. Louis, MO, United States) was measured according to a method described by Lall et al. (2013). Detached cells were seeded in 96-well plates (100 µL) at a concentration of 1.0 × 10^5^ cells/well. Plates were incubated overnight at 5% CO_2_ and 37°C to allow for cell adherence. Mupirocin (stock concentration of 80 mM (40 mg/ml) in DMSO) was serially diluted in DMEM media in a 24-well plate to obtain concentrations ranging from 5.0 × 10^−2^−1.60 mM (25–800 μg/ml). Serially diluted sample was transferred (100 µL) to the 96-well plates, containing 100 µL of cells, at final test concentrations ranging between 2.5 × 10^–2^ - 0.8 mM (12.5–400 μg/ml). A media (untreated) control, 1% DMSO (vehicle) control, 0% control (no cells) and a positive control (actinomycin D, purity ≥95%) (Sigma Chemicals Co., St. Louis, MO, United States), at final concentration ranging from 3.1 × 10^–4^—0.04 µM (3.9 × 10^–4^—0.05 μg/ml), were included. Plates were incubated for 72 h, where after 20 µL of PrestoBlue^®^ cell viability reagent (ThermoFisher Scientific, Johannesburg, South Africa) was added to each well and incubated for a further 2 h. Fluorescence was measured at an excitation/emission of 560/590 nm using a Victor Nivo microplate reader (Perkin Elmer Inc., Massachusetts, United States). Mupirocin and the controls were tested in triplicate in three independent experiments (*n* = 3). Percentage cell viability was calculated using the following equation. The fifty percent inhibitory concentrations (IC_50_) were determined using GraphPad Prism 4 software.

$$\%\;Cell\;viability=\frac{Flour.\;\;sample-Flour\;0\%\;control}{Flour.\;DMSO\;control-Flour\;0\%\;control}\times 100$$

### 2.3 Wound Healing

The scratch assay was performed according to a method described by Liang et al. (2007), with modifications. The HaCat cells were seeded at a concentration of 1.5×10^5^ cells/mL in a 24-well plate and incubated overnight, at 37°C and 5% CO_2_, to form a confluent monolayer. Thereafter a p1000 pipette tip was used to scratch a cross in the middle of the well to simulate a wound. Cell debris was removed by aspirating the media and adding fresh complete media. Thereafter, cells were treated with mupirocin, at final concentrations of 0.1 and 0.2 mM (50 and 100 μg/ml) for 18 h. Controls included cells grown in media (untreated) and cells treated with 0.25% DMSO (vehicle control). Mupirocin and the controls were tested in duplicate in three independent experiments (*n* = 3). Images were captured using a light microscope at 4 × magnification (Zeiss Primovert, Carl Zeiss (Pty) Ltd., Johannesburg, South Africa) directly after the initial scratch was made (0 h) and 18 h after treatment. The percentage wound closure was measured using the ImageJ 1.5 imaging software (National Institute of Health (NIH), United States) with the following steps: Images were converted to 8-bit and a bandpass filter was applied. Thereafter the threshold was adjusted and a radius filter was applied (between 7 and 10). The wand tool was then used to select the boarder of the scratch and measured to record the area, where after the % closure calculated using the following equation:

$$Wound\;closure\;\left(\%\right)=\;\frac{Area\;of\;scratch\;\left(0h\right)-Area\;of\;scratch\;\left(18h\right)}{Area\;of\;scratch\;\left(0h\right)}\times 100$$

### 2.4 Growth Factor Quantification

The quantification of the human growth factors was performed using the LEGENDPlex™ Human Growth Factor Panel Kit (Biocom Biotech, Centurion, South Africa, Cat # 740180). Cell free supernatant was collected from the24-well plate used in the scratch assay, after the 18 h images were taken by centrifuging the plates at 980 rpm for 5 min, and transferring the supernatant to sterile 96-well plates, which were stored at −80°C until further use. To the remaining cells in the 24-well plate, PrestoBlue^®^ cell viability reagent was added and incubated for a further 2 h, after which fluorescence and percentage viability was determined as described in *Wound healing Section*. The quantification of growth factors was performed according to the manufacturer’s protocol. Briefly, 25 µL of sample and standards were added to the supplied v-bottom plate, followed by the addition of 25 µL assay buffer, 25 µL pre-mixed beads and 25 µL detection antibodies to each well. The standards were tested at two concentration ranges; 0–10,000 pg/ml (EGF, M-CSF and SCF) and 0–50,000 pg/ml (Ang-2, EPO, FGF-basic, G-CSF, GM-CSF, HGF, PDGF-AA, PDGF-BB, TGF-α and VEGF). The plate was then covered with foil and placed on a shaker for 2 h at 25°C. After 2 h, 25 µL streptavidin-phycoerythrin (SA-PE) conjugate was added to all the wells, and the plate was further incubated on a shaker. After 30 min, the plate was centrifuged at 4000 rpm for 5 min, where after the supernatant was removed and the plate was washed using 200 µL of the supplied wash buffer (1 × wash buffer). The beads were re-suspended in 150 µL 1 × wash buffer and the samples were read on a BD Accuri™ C6 Plus flow cytometer (BD Biosciences, San Diego, CA, United States), acquiring between 2000–2,500 events. Data was analysed using the LEGENDPlex™ v8.0 software to determine the concentration of growth factors (pg/ml). Samples were tested in triplicate (*n* = 3).

### 2.5 Statistical Analysis

All data is represented as mean ± standard deviation (unless otherwise specified in the footnotes). Number of replicates and independent experiments are described in the methods section. The IC_50_ values were calculated using sigmoidal dose-response curves and non-linear regression analysis with constraints set at 100 (top) and 0 (bottom) using GraphPad Prism Version 4.0 software. Statistical analysis was performed using one-way analysis-of-variance (ANOVA) followed by Tukey’s multiple comparison test. Statistical significance was displayed as ∗*p* < 0.05, ∗∗*p* < 0.01 and ∗∗∗*p* < 0.001 compared to the control (+).

## 3 Results

### 3.1 Effect of Mupirocin on Human Keratinocytes (HaCat)

**FIGURE 2:**
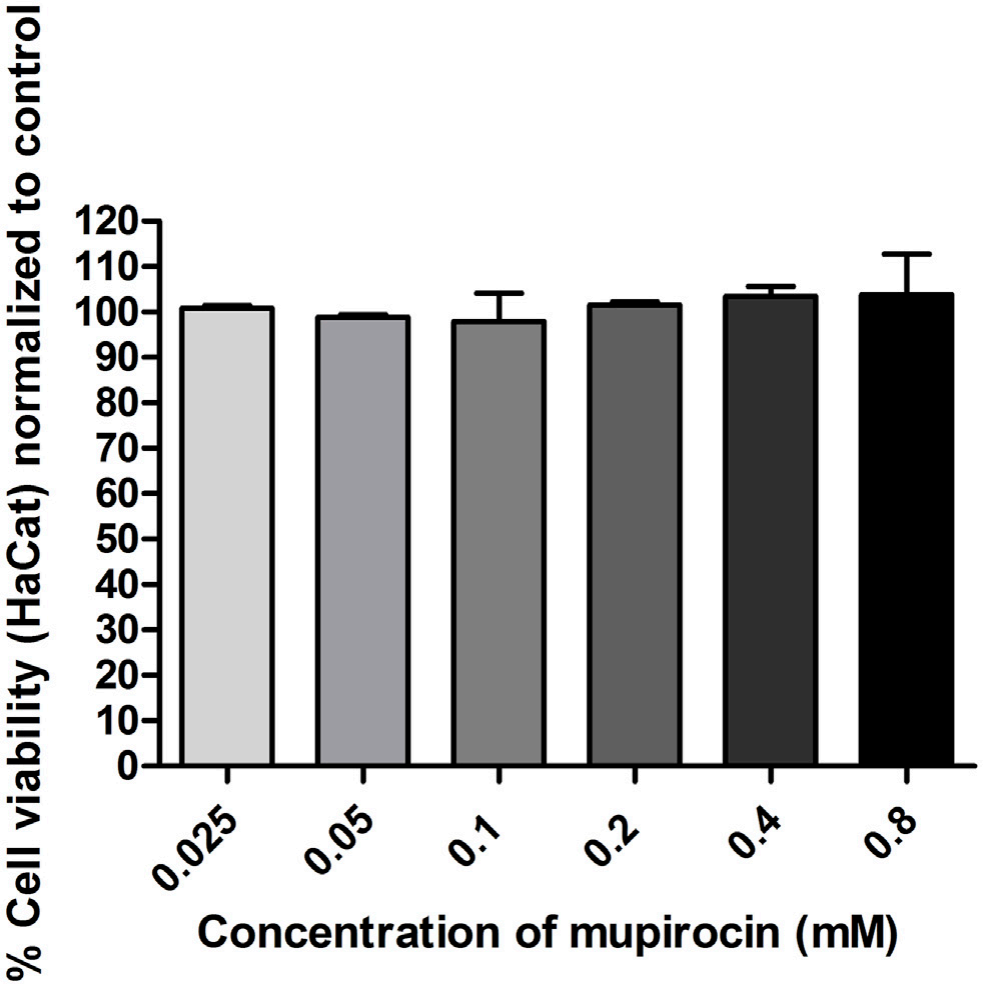
Percentage normalized cell viability of human keratinocytes (HaCat) treated with mupirocin, at concentrations ranging between 2.5 × 10^−2^−0.8 mM, after 72 h. Data is presented as mean ± SEM of two independent experiments conducted in triplicate.

The cytotoxicity of mupirocin was evaluated against HaCat cells after 72 h treatment. Mupirocin showed low to no toxicity against HaCat cells (IC_50_ value >0.8 mM; > 400 μg/ml) (Figure 2). The cytotoxicity of mupirocin was compared to the positive toxic inducer, actinomycin D, which showed an IC_50_ value of 5.6 × 10^–3^ ± 2.4×10^–4^ μM (7.0 × 10^–3^ ± 3.0×10^–4^ μg/ml).

### 3.2 Mupirocin Induced Wound Healing in Human Keratinocytes

**FIGURE 3:**
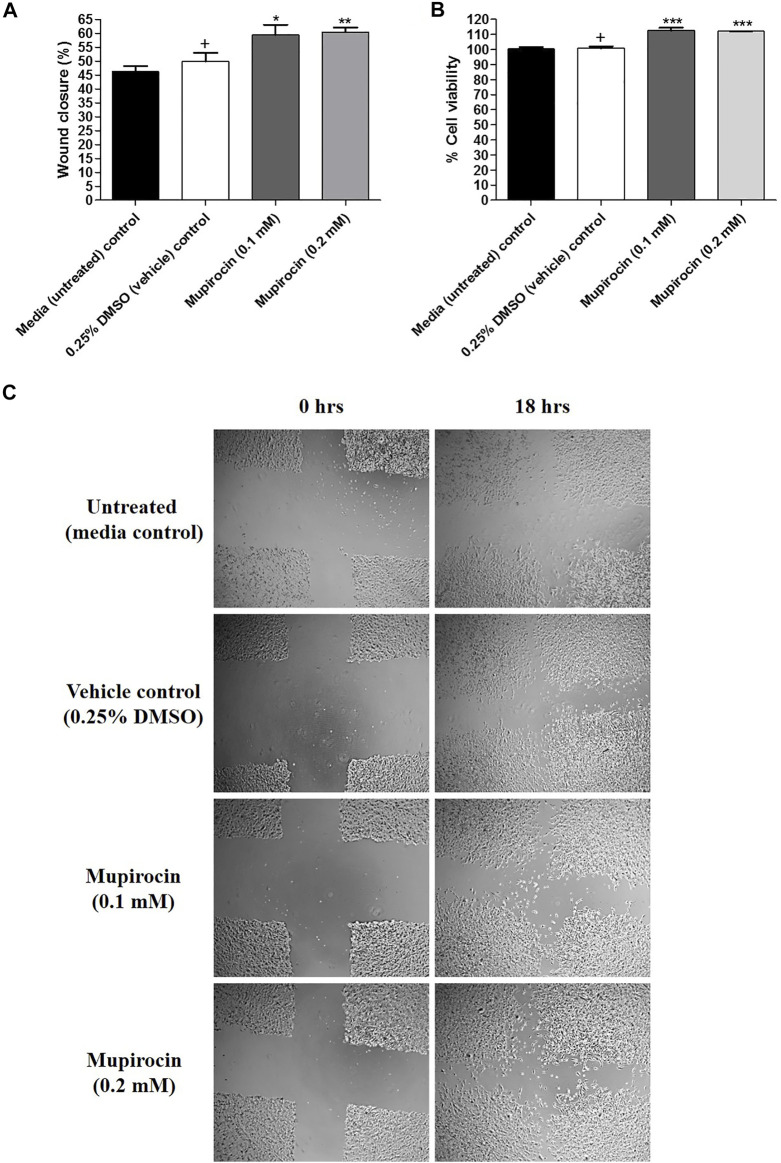
**(A)** Percentage (%) wound closure, **(B)** percentage (%) cell viability and **(C)** light microscope images (4 x magnification) representing the percentage (%) wound closure of human keratinocytes (HaCat) treated with mupirocin (at 0.1 and 0.2 mM) after 18 h. Controls included cells grown in media (untreated) and cells treated with 0.25% DMSO (vehicle control). Data shown are mean ± SD (*n* = 3). *p < 0.05, **p < 0.01 and ***p < 0.001 indicates statistical significance when compared to the 0.25% DMSO vehicle control (+). Statistical analysis was done using one-way ANOVA followed by Tukey’s multiple comparison test.

The wound healing potential of mupirocin was assessed at non-toxic concentrations (0.1 and 0.2 mM; 50 and 100 μg/ml), which correlated with low levels (MIC) of *S. aureus* resistance to mupirocin. Wound healing was compared to the proliferation of cells under normal conditions (untreated media control) and vehicle treated control cells (Figure 3).

After exposure to mupirocin at 0.1 and 0.2 mM for 18 h, wound healing significantly increased with a percentage wound closure of 59.38 ± 3.70% (*p* < 0.05) and 60.35 ± 1.74% (*p* < 0.01), compared to the vehicle control (49.84 ± 3.14%). In addition, there was no significant difference in wound closure of untreated cells (46.30 ± 1.89%) compared to the vehicle control (Figure 3A). It was further noted, that although mupirocin at 0.2 mM showed higher wound closure than at 0.1 mM there was no significant difference in wound closure when the two concentrations of mupirocin were compared to each other.

Furthermore, cell viability measured from the scratch assay, showed that there was no statistical difference between the untreated control (100.37 ± 1.20%) and the vehicle control (100.78 ± 1.34%), however when mupirocin was compared to the vehicle control, a significant difference (*p* < 0.001) was noted at 0.1 mM (112.59 ± 1.95%) and 0.2 mM (112.13 ± 0.14%), with increased cell proliferation, which correlated with the increase in wound closure (Figure 3B).

### 3.3 Mupirocin Increased Growth Factor Production in Human Keratinocytes

**FIGURE 4:**
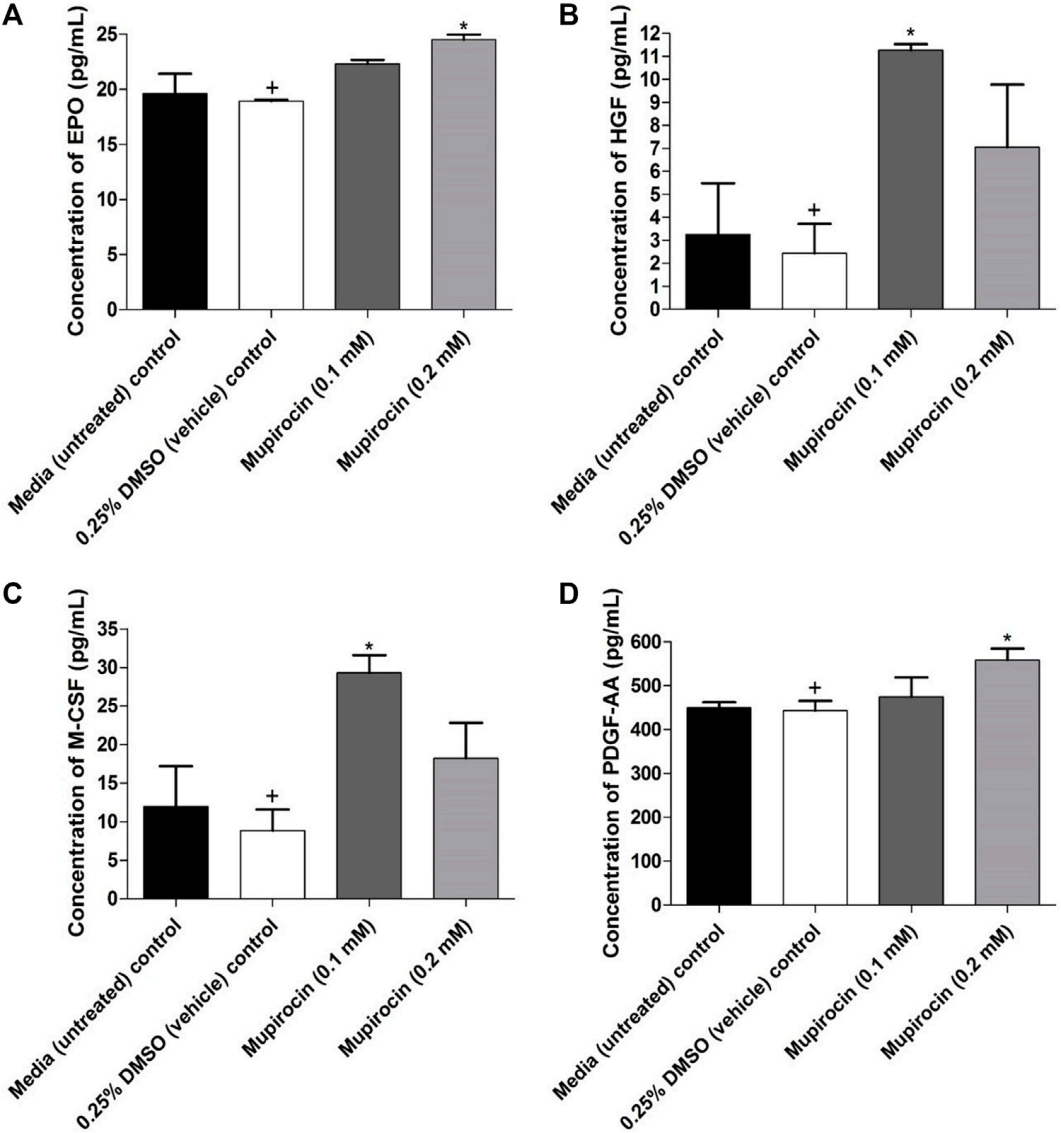
Concentration (pg/ml) of **(A)** erythropoietin (EPO), **(B)** hepatocyte growth factor (HGF), **(C)** macrophage colony-stimulating factor (M-CSF) and **(D)** platelet derived growth factor AA (PDGF-AA) expressed by human keratinocytes (HaCat) treated with mupirocin (at 0.1 and 0.2 mM) after 18 h. Controls included cells grown in media (untreated) and cells treated with 0.25% DMSO (vehicle control). Data shown are mean ± SD (*n* = 3). *p < 0.05 indicates statistical significance when compared to the 0.25% DMSO vehicle control (+). Statistical analysis was done using one-way ANOVA followed by Tukey’s multiple comparison test.

Cell-free supernatant was collected to determine the effect of mupirocin on growth factor production in HaCat cells. Untreated cells produced EPO, HGF, M-CSF and PDGF-AA, whereas no production of angiopoietin-2 (Ang-2), epidermal growth factor (EGF), fibroblast growth factor (FGF)-basic, granulocyte-colony stimulating factor (G-CSF), granulocyte-macrophage colony-stimulating factor (GM-CSF), platelet-derived growth factor (PDGF)-BB, stem cell factor (SCF), transforming growth factor (TGF)-α and vascular endothelial growth factor (VEGF) was observed. Furthermore, the vehicle control (HGF: 2.43 ± 1.28 pg/ml, M-CSF: 8.85 ± 2.73 pg/ml, EPO: 18.93 ± 0.12 pg/ml and PDGF-AA: 443.48 ± 21.88 pg/ml) did not show a significant difference when compared to the untreated control (HGF: 3.26 ± 2.23 pg/ml, M-CSF: 11.97 ± 5.22 pg/ml, EPO: 19.62 ± 1.79 pg/ml and PDGF-AA: 449.54 ± 12.69 pg/ml), for each of the evaluated growth factors (Figure 4). At 0.1 mM, mupirocin showed a significant (*p* < 0.05) increase in the production of HGF (11.27 ± 0.26 pg/ml) and M-CSF (29.31 ± 2.30 pg/ml) compared to the vehicle control, whereas no significant difference was noted at 0.2 mM for both HGF (7.06 ± 2.72 pg/ml) and M-CSF (18.21 ± 4.60 pg/ml) (Figures 4B,C). However, mupirocin at 0.2 mM showed a significant increase (*p* < 0.05) in the production of EPO (24.51 ± 0.46 pg/ml) and PDGF-AA (558.15 ± 26.73 pg/ml), compared to the vehicle control, whereas mupirocin at 0.1 mM did not show a significant difference in EPO (22.32 ± 0.35 pg/ml) and PDGF-AA (474.94 ± 43.90 pg/ml) production (Figures 4A,D). However, when the increase in growth factor production of mupirocin at 0.1 mM was compared to the production of mupirocin at 0.2 mM, no statistical difference was observed, indicating that mupirocin at 0.1 and 0.2 mM showed similar effects on growth factor production (Figure 4), which correlated with the wound healing activity and cell viability at both concentrations (Figure 3).

## 4 Discussion

Mupirocin was evaluated for its potential to promote wound healing by stimulating the proliferation of human keratinocytes. Mupirocin showed a significant increase in HaCat cell proliferation, as investigated in the scratch assay (Figure 3) and showed low to no toxicity against HaCat cells when tested at a concentration of 0.8 mM (Figure 2), according to thresholds set by (Kuete and Efferth, 2015). This was further confirmed by the significant increase in cell proliferation observed at a concentration of 0.1 and 0.2 mM (Figure 3B).

In a similar study by Kamlungmak et al. (2021), mupirocin, a 2% (w/w) mupirocin ointment and a mupirocin nanoparticle loaded hydrogel (MLH), each tested at a concentration of 500 μg/ml, were considered non-toxic to HaCat cells and human fibroblasts (BJ) after 24 h exposure, with cell viability above 80% for each sample. Furthermore, the rate of wound closure was observed in BJ cells, after creation of a scratch, after 24, 48 and 72 h. The mupirocin ointment showed the least wound closure, which was lower than that of the untreated control, whereas mupirocin and the MLH showed similar wound closure rated to that of the untreated control, therefore not having a significant effect of fibroblast proliferation and migration. However, in this study they found that mupirocin significantly enhanced the production of TNF-α in macrophages, which plays a significant role in the early inflammatory stage of wound healing. In the current study, significant wound closure was observed in keratinocytes, suggesting that mupirocin may enhance wound closure through keratinocytes rather than fibroblasts, and also plays a role in the inflammatory stage, as observed by (Kamlungmak et al., 2021).

In the current study it was further found that the increase in cell proliferation, leading to wound closure, was due to increased production of EPO, HGF, M-CSF and PDGF-AA by keratinocytes (Figure 4). The significant role of M-CSF in wound healing was displayed in a study by Li et al. (2016) where wound size in mice significantly decreased after five and ten day topical application with 2 ng/0.1 ml M-CSF, whereas, the application of 1 µg/0.1 ml M-CSF neutralizing antibody significantly decreased wound healing. Similarly, Sorg et al. (2009), reported that a single high dose of EPO (5000 U/kg body weight) (SHD-EPO) on the day of wounding in SKH-1-*hr* hairless mice, significantly accelerated wound healing after day six of application. In addition SHD-EPO, significantly increased the migration of both fibroblasts and keratinocytes and induced maturation of newly formed microvascular networks, thereby accelerating wound healing. A follow up study by Siebert et al. (2011), reported that a single injection of EPO (5000 U/kg body weight) at the day of wounding in SKH-1-*hr* hairless mice significantly increased wound epitheliazation at day 3, 9 and 12. The wound healing effect of EPO has been reported to act via the transforming growth factor (TGF-*β*), which plays a major role in cell proliferation of fibroblasts and keratinocytes, re-epithelization and angiogenesis (Werner and Grose, 2003). A study by Li et al. (2013), showed that HGF accelerates wound healing, both *in vitro* and *in vivo*, by increasing the expression of the cell adhesion molecule, *β* 1-integrin and the cytoskeleton remodeling protein integrin-linked kinase (ILK), in rat epidermal cells. HGF has been reported to induce cell division of keratinocytes, melanocytes and epithelial cells, stimulates angiogenesis and induces wound healing (Jiang and Hiscox, 1997). In addition, platelets are one of the first cell types to respond after wound formation, and are responsible for releasing several growth factors, including PDGF, which in turn attract neutrophils and monocytes to the wound site, initiating the inflammatory phase of wound healing (Park et al., 2017). Keratinocytes, endothelial cells and fibroblasts have further been reported to produce PDGF (Kiwanuka et al., 2012).

Although in the current study, the effect of mupirocin on cell migration was not evaluated, previous studies have shown that EPO, HGF, M-CSF and PDGF-AA play significant roles in stimulating cell migration in the wound healing process. This suggest that, mupirocin, which increases the proliferation of keratinocytes and enhances the production of these growth factors, may also have an effect on cell migration, however this will need to be evaluated in future studies such as through a transwell migration assay or including mitomycin C as an inhibitor of cell proliferation, thereby excluding the effect of proliferation on wound closure (Varankar and Bapat, 2018).

Due to increased resistance of wound pathogens to antibiotics, there is a growing interest in combining antibiotics to combat drug resistant bacteria through their synergistic effect (Worthington and Melander, 2013). Therefore, an alternative mechanism to mitigate the further emergence of resistance could be to combine mupirocin with another antibiotic, thereby enhancing the antibacterial effect through synergistic activity, while enhancing the wound healing activity through the increased production of growth factors in keratinocytes by mupirocin. A study by Thangamani et al. (2015), reported the activity of simvastatin against MRSA, with an MIC_50_ of 32 μg/ml, the inhibition of *S. aureus* toxins, Panton-Valentine leucodin (PVL) and *α*-hemolysin (Hla), the reduction in *S. aureus* biofilms and the decrease in bacterial counts of MRSA in infected mice by 75 and 90% at 1 and 3% simvastatin, respectively. Furthermore, when combined with mupriocin, a synergistic effect was observed against *S. aureus* clinical isolates.

In conclusion, mupirocin, plays a significant role in wound healing by increasing proliferation of human keratinocytes and enhancing the production of several growth factors. Previous studies have also shown that mupirocin enhances the production of TNF-α, in macrophages, in the early stages of wound healing, thereby contributing towards the inflammatory phase. Furthermore, mupirocin should be evaluated for its potential to stimulate migration of keratinocytes using a migration assay and whether it is able to stimulate angiogenesis, a crucial role required for wound healing, using an *in vivo* assay such as the *ex ovo* chorioallantoic membrane assay, where the effect of mupirocin on vascularization can be observed. Furthermore, due to the decline in the discovery of new antibiotics, mupirocin should be evaluated in combination with other antibiotics to determine whether there is enhanced activity against mupirocin resistant *S. aureus* strains (synergistic activity) while potentially showing enhanced wound healing activity.

## Data Availability

The raw data supporting the conclusions of this article will be made available by the authors, without undue reservation.
